# Genome-wide array-CGH analysis reveals *YRF1* gene copy number variation that modulates genetic stability in distillery yeasts

**DOI:** 10.18632/oncotarget.5594

**Published:** 2015-09-10

**Authors:** Anna Deregowska, Marek Skoneczny, Jagoda Adamczyk, Aleksandra Kwiatkowska, Ewa Rawska, Adrianna Skoneczna, Anna Lewinska, Maciej Wnuk

**Affiliations:** ^1^ Department of Genetics, University of Rzeszow, Rzeszow, Poland; ^2^ Department of Genetics, Institute of Biochemistry and Biophysics, Polish Academy of Sciences, Warsaw, Poland; ^3^ Laboratory of Mutagenesis and DNA Repair, Institute of Biochemistry and Biophysics, Polish Academy of Sciences, Warsaw, Poland; ^4^ Department of Biochemistry and Cell Biology, University of Rzeszow, Poland

**Keywords:** distillery yeasts, genome, array-CGH, chromosomes, genetic instability, Chromosome Section

## Abstract

Industrial yeasts, economically important microorganisms, are widely used in diverse biotechnological processes including brewing, winemaking and distilling. In contrast to a well-established genome of brewer's and wine yeast strains, the comprehensive evaluation of genomic features of distillery strains is lacking. In the present study, twenty two distillery yeast strains were subjected to electrophoretic karyotyping and array-based comparative genomic hybridization (array-CGH). The strains analyzed were assigned to the *Saccharomyces sensu stricto* complex and grouped into four species categories: *S. bayanus*, *S. paradoxus*, *S. cerevisiae* and *S. kudriavzevii*. The genomic diversity was mainly revealed within subtelomeric regions and the losses and/or gains of fragments of chromosomes I, III, VI and IX were the most frequently observed. Statistically significant differences in the gene copy number were documented in six functional gene categories: 1) telomere maintenance *via* recombination, DNA helicase activity or DNA binding, 2) maltose metabolism process, glucose transmembrane transporter activity; 3) asparagine catabolism, cellular response to nitrogen starvation, localized in cell wall-bounded periplasmic space, 4) siderophore transport, 5) response to copper ion, cadmium ion binding and 6) L-iditol 2- dehydrogenase activity. The losses of *YRF1* genes (Y' element ATP-dependent helicase) were accompanied by decreased level of Y' sequences and an increase in DNA double and single strand breaks, and oxidative DNA damage in the *S. paradoxus* group compared to the *S. bayanus* group. We postulate that naturally occurring diversity in the *YRF1* gene copy number may promote genetic stability in the *S. bayanus* group of distillery yeast strains.

## INTRODUCTION

The budding *Saccharomyces cerevisiae* is the most scientifically and industrially exploited species among the *Saccharomyces sensu stricto* complex as it is widely used as a model organism and in the fermentation processes such as the production of food and alcoholic beverages [1, 2]. There are at least seven natural *Saccharomyces sensu stricto* species (*S. cerevisiae*, *S. paradoxus*, *S. mikatae*, *S. kudriavzevii*, *S. arboricola*, *S. eubayanus* and *S. uvarum*) and numerous related industrial hybrids of a biotechnological interest (e.g., *S. cerevisiae* x *S. kudriavzevii*, *S. pastorianus, S. bayanus, S. cerevisiae* x *S. mikatae)* [1, 3–11]. More recently, *S. paradoxus* has been also established as a main yeast component in Croatian wines that may suggest a potentially important enological characteristics for this species [12].

The domestication within the *Saccharomyces sensu stricto* complex has led to the evolution of special phenotypic features *via* hybridization, polyploidization, gene duplication and gene transfer [2]. The best example of how fermentative conditions can shape the yeast genome is the acquiring *SSU1-R* allele-based resistance to sulfite by wine yeasts [13]. This adaptation is a result of a reciprocal translocation between chromosomes VIII and XVI due to unequal crossing-over mediated by microhomology between very short sequences on the 5′ upstream regions of the *SSU1* and *ECM34* genes that provokes the induction of the SSU1 transporter and increases the ability of yeast cells to expulse sulfite from the cytoplasm [13]. This genetic change can be found in 50% of the wine strains, whereas it has not been observed among wild strains suggesting that the use for millennia of sulfite as a preservative in wine production could have favored its selection [14].

In contrast to the best studied genomes of wine and brewing yeast strains, the information on genetic and genomic diversity of yeast isolates involved in the production of distilled spirits is limited. In the present study, array-CGH-based genome-wide analysis of twenty two commercially available distillery yeasts was conducted. We have revealed four groups with different pattern of the gene copy number variants that in the case of the *YRF1* gene dosage diversity may provoke changes in genetic stability.

## RESULTS

### Electrophoretic karyotyping of distillery yeasts reveals four species categories

As there are limited number of published data on genomic and genetic characteristics of distillery yeasts [15, 16], the karyotype and the genome of, commercially available and widely used in food industry, twenty two distillery yeast strains were comprehensively investigated (Table 1).

**Table 1:** Distillery yeast strains used in this study

No.	Trade name	Company
1	Samogon turbo	CBF Drinkit
2	Superyeast T48 Dual Use	CBF Drinkit
3	Spiritferm Extreme 8 kg Turbo	Spiritferm
4	Spiritferm T3	Spiritferm
5	Spiritferm turbo fruit	Spiritferm
6	Spiritferm Moskva style	Spiritferm
7	Coobra 24 Snabbsats	CBF Drinkit
8	Coobra 6 Magnum Snabbsats	Vendor
9	Coobra 8 Snabbsats	Vendor
10	Coobra 48 Turbo Yeast	CBF Drinkit
11	Coobra RUM YEAST	CBF Drinkit
12	Double Snake Turbo Yeast C3 Extra	Hambleton Bard Ltd.
13	Alcotec Pure Turbo Super Yeast 48	Hambleton Bard Ltd.
14	Drożdże gorzelnicze Turbo 72h	BIOWIN
15	Black Bull Turbo Yeast	Avedore Trading
16	Gozdawa 1410 Turbo	Gozdawa
17	Superyeast T Vodka Star	CBF Drinkit
18	Alcotec Vodka Star Turbo Yeast	Hambleton Bard Ltd.
19	Alcotec Single Strain Whisky with Amyloglucosidase	Hambleton Bard Ltd.
20	Fermiol drożdże gorzelnicze	BIOWIN/FERMIOL
21	BIOWIN Turbo Super Yeast 48h	BIOWIN
22	Alcotec Pure Turbo Super Yeast 24h	Hambleton Bard Ltd.

**Figure 1:**
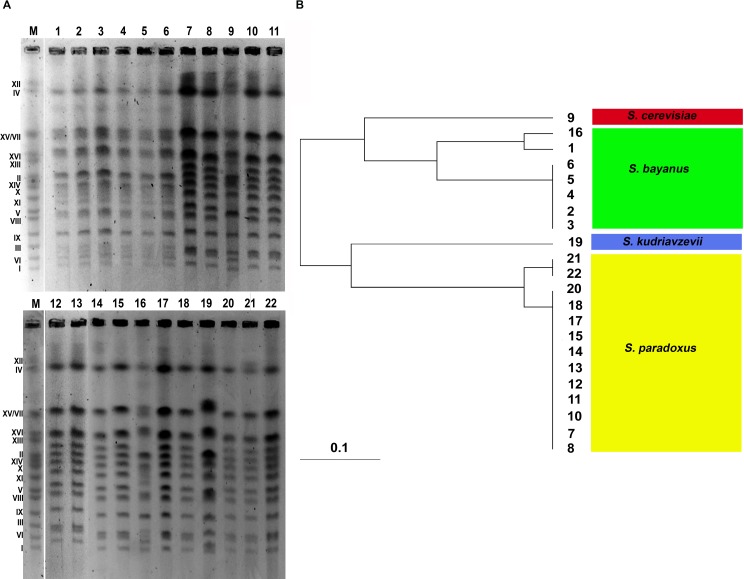
Electrophoretic karyotyping of twenty two distillery yeast strains (A, lanes from 1 to 22) The yeast *S. cerevisiae* chromosome marker YNN295 (BIORAD) is shown (**A.**, lane M). The dendrogram of chromosome band-based similarity is also presented **B.** The species classification within the *Saccharomyces sensu stricto* complex is provided.

On the basis of PFGE separation (electrophoretic karyotyping), one can conclude that all yeasts examined belonging to the *Saccharomyces sensu stricto* complex [17]. In general, the chromosome number of analyzed yeasts is 16 (Figure 1). However, an additional band was observed between chromosomes IV and VII in strains from 1 to 6 and strain 16 and between chromosomes I and VI in strain 19 (Figure 1). In almost all strains examined, chromosomes IV and XII migrated together (Figure 1).

The strains from 1 to 6 and strain 16 had the *S. bayanus*-like chromosome pattern, whereas strains 7, 8, 10, 11, 12, 13, 14, 15, 17, 18, 20, 21 and 22 were classified as *S. paradoxus*, strain 9 as *S. cerevisiae* and strain 19 as *S. kudriavzevii* (Figure 1). A chromosomal band of about 1300 kb (between chromosomes IV and VII) observed in strains from 1 to 6 and strain 16 is a characteristic feature of *S. bayanus* karyotype [18]. Chromosome similarity between analyzed strains was also further evaluated using UPGMA clustering (Figure 1). Strains from 2 to 6 were the most similar within assigned *S. bayanus* group, whereas strains 1 and 16 differed from other *S. bayanus* strains (Figure 1). Similarly, strains 21 and 22 were more distant from other *S. paradoxus* strains (Figure 1).

### Distillery yeasts are diploid

**Figure 2:**
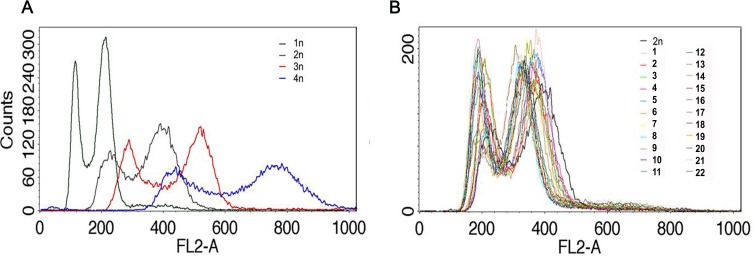
The ploidy analysis Fluorescence-activated cell sorting (FACS)-based analysis of DNA content of distillery strains **B.** Haploid, diploid, triploid and tetraploid reference strains are also shown **A.**

The ploidy of distillery strains was then analyzed using fluorescence-activated cell sorting (FACS) (Figure 2).

We found that all strains used were diploid when compared to reference laboratory yeast cells with known ploidy (haploid, diploid, triploid and tetraploid cells) (Figure 2).

### The diversity of gene copy number and loci-specific gains and losses involve mainly the subtelomeric regions

**Figure 3:**
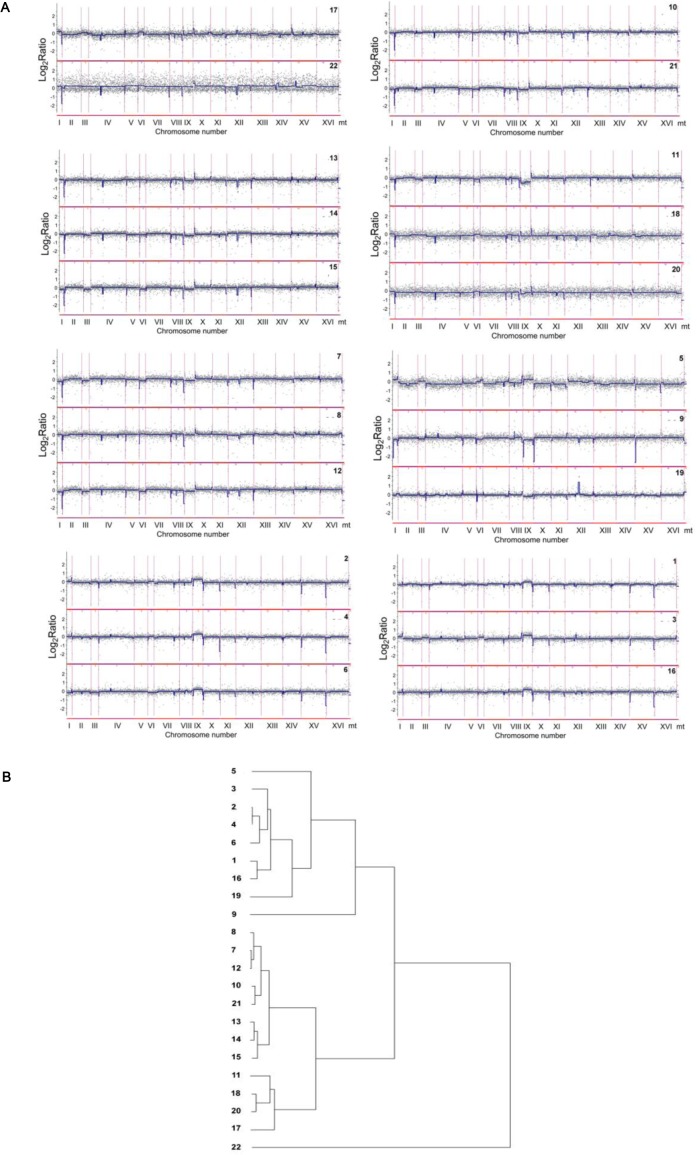
Comparison of the gene copy number between analyzed distillery yeasts using array-CGH **A.** The strains with similar array-CGH profiles were grouped together. Each grey dot represents the value of the log_2_ ratio for an individual gene. Blue lines were provided to emphasize the most accented differences (DNA losses and gains). **B.** The relatedness of distillery strains as determined by cluster analysis. Similarity tree is shown (see Materials and Methods section for the details).

After electrophoretic karyotyping, the genome of distillery strains was characterized using array-based comparative genomic hybridization (array-CGH) (Figure 3).

**Figure 4:**
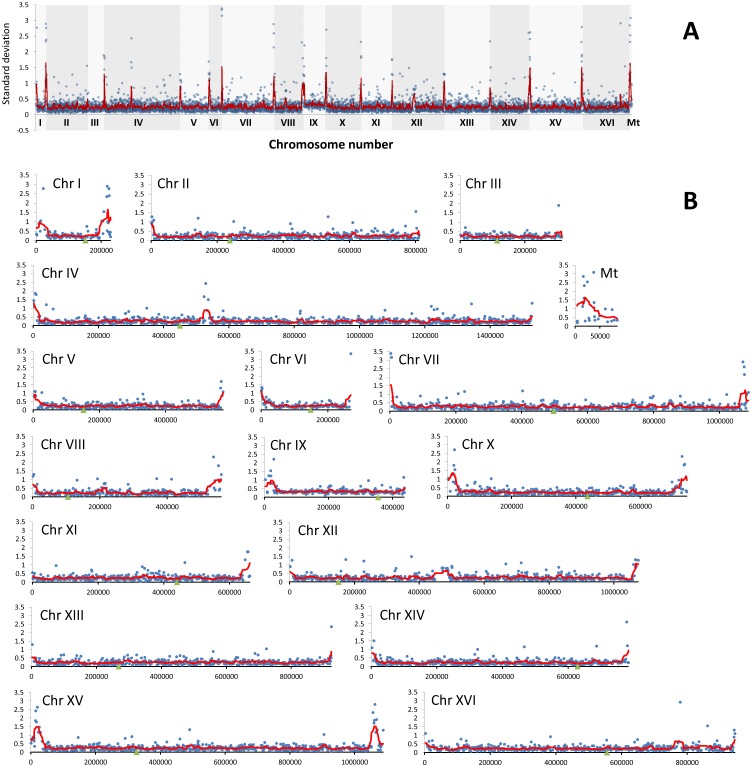
The divergence of relative abundance of genes as determined by array-CGH analysis represented by standard deviation (SD) of log_**2**_ ratio values for each gene in all analyzed strains **A.** The summary plot for the whole genome. **B.** Individual plots for each chromosome. Blue dots indicate the SD values for individual genes, the red line denotes the smoother trend calculated by moving average of SD values to expose the genome regions of higher log_2_ ratio divergence and green triangles indicate centromere position.

The analysis of array-CGH profiles revealed variabilities in the gene copy number exclusively within the subtelomeric regions of all analyzed chromosomes and two short intrachromosomal regions of chromosomes IV and XII (Figures 3A and 4).

The differences between strains were more accented including the losses and/or gains of fragments of chromosomes I, III, VI and IX, and in the case of strain 5 also the changes within chromosome XII (Figure 3A). The gain of chromosomes I and VI in strains 3, 5 and 17, and the loss of chromosomes I and VI in strains 7, 9, 11, 12 and 15 were revealed (Figure 3A). The gain of chromosome III in strains 3, 5 and 17, and the loss of chromosome III in strains 7, 9, 11, 12, 14, 15 and 21 were observed (Figure 3A). The most variable chromosome was chromosome IX. The gains of chromosome IX were shown in strains 1, 2, 3, 4, 5, 6 and 16, whereas the losses of chromosome IX were documented in strains 7, 9, 11, 12, 15 and 19 (Figure 3A). The gains of chromosome XII was exclusively reported in strain 5. Interestingly, small chromosomes were frequently affected and changes in one small chromosome were accompanied by changes in other small chromosomes. However, these gains and losses were too small to be interpreted as duplications or deletions of chromosomal regions or whole chromosome aneuploidy events within the whole population of particular strain. Perhaps, the chromosome variations may suggest the cellular heterogeneity within a population. Additionally, array-CGH profiles were used to estimate the level of similarity (relatedness) between distillery strains on the basis of observed diversity in subtelomeric regions and chromosome IX (Figure 3B). Array-CGH-based relationships between analyzed strains were comparable with electrophoretic karyotyping-based relationships (Figures 1 and 3B). The strains from 1 to 6 and strain 16 already classified as *S. bayanus* (Figure 1) were clustered together (Figure 3B). According to both similarity analyses used, strains 2, 4 and 6, and strains 1 and 16 were closely located (Figures 1 and 3B). The strains belonging to *S. paradoxus* species (Figure 1), were grouped into several categories using array-CGH-based analysis, namely the group of the strains 7, 8 and 12; 10 and 21; 13, 14 and 15; 11, 17, 18 and 20 (Figure 3B). The most variable was strain 22 (*S. paradoxus* species, Figure 1) with its own category (Figure 3B).

### Gene ontology overrepresentation profiles are species-specific

**Figure 5:**
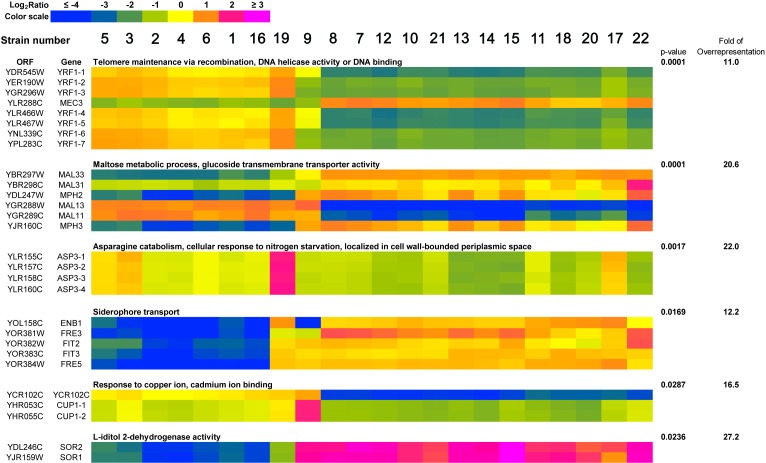
A heat map generated from array-CGH data Functional categories overrepresented in the group of genes that were the most divergent among analyzed strains are shown. The strains were ordered according to the result of clustering analysis (Figure 3B) and the selected genes were grouped according to their functional assignment. Positive and negative log_2_ ratio values represent higher and lower than average abundance of the gene, as determined by array-CGH analysis (see Materials and Methods section for the details).

As the observed differences in the gene copy number and loci-specific gains and losses may affect the functional properties of distillery strains, the genes that were most divergent according to array-CGH-based analysis were then subjected to gene ontology overrepresentation analysis (Figure 5).

The selected gene-set consisted of 257 genes, for which, in at least one strain, the log_2_ ratio value was greater than four standard deviations of log_2_ ratio calculated for all genes in all strains. Six functional categories overrepresented in the group of selected genes were revealed, namely 1) telomere maintenance *via* recombination, DNA helicase activity or DNA binding; 2) maltose metabolism process, glucose transmembrane transporter activity; 3) asparagine catabolism, cellular response to nitrogen starvation, localized in cell wall-bounded periplasmic space; 4) siderophore transport; 5) response to copper ion, cadmium ion binding and 6) L-iditol 2- dehydrogenase activity (*p* < 0.05) and are presented as a heat map in Figure 5. Species-dependent variability in the gene copy number within functional categories of selected genes were revealed, e.g., similar genetic features were observed among strains belonging to *S. bayanus* species that differed from genetic features in the strains of *S. paradoxus* species (Figure 5). Moreover, strains 9 (*S. cerevisiae*) and 19 (*S. kudriavzevii*) had their own overrepresentation profiles (Figure 5). Interestingly, within functional category of genes involved in the telomere maintenance *via* recombination, DNA helicase activity or DNA binding, the gains of *YRF1* genes (helicases encoded by the Y' element of subtelomeric regions) were exclusively shown in the *S. bayanus* strain group and strain 19 (*S. kudriavzevii*), whereas the losses of *YRF1* genes were observed in the *S. paradoxus* strain group (Figure 5). A heat map generated from array-CGH data reflecting the variability in the gene copy number of the whole genome of all analyzed distillery strains is also presented in Supplementary File 1.

### The *YRF1* gene copy number corresponds to the presence of Y' telomeric sequences

**Figure 6:**
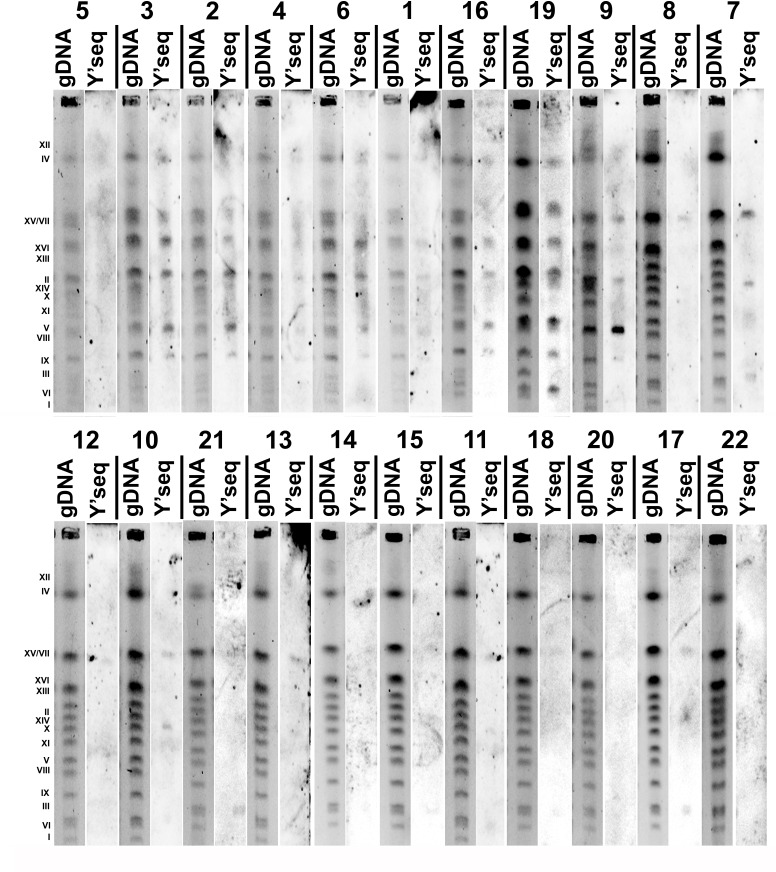
The presence of Y' telomeric sequences in twenty two distillery yeasts (categories from 1 to 22, lane gDNA: chromosome pattern of an individual strain, lane Y' seq: Y' telomeric sequences) detected using Southern blot using Y' telomeric sequence probes

Since array-CGH-based analysis revealed that the majority of genomic differences can be found in subtelomeric regions of the genome of distillery strains and Y' element ATP-dependent helicase activity may be affected in the opposite direction in the *S. bayanus* and *S. paradoxus* strain groups (Figures 3, 4 and 5), we then evaluated the presence of Y' telomeric sequences in all examined strains (Figure 6).

Y' telomeric sequences were the most accented in the *S. bayanus* strain group, whereas they were marginally noticeable in the *S. paradoxus* strain group (Figure 6). Southern blot data using Y' telomeric probes are in agreement with array-CGH results (Figure 5). The same relationship was observed for strain 19 (*S. kudriavzevii*) with the highest log_2_ ratios of *YRF1* genes (Figure 5) and rich in Y' telomeric sequences (Figure 6).

### The *YRF1* gene copy number modulates genetic stability

**Figure 7:**
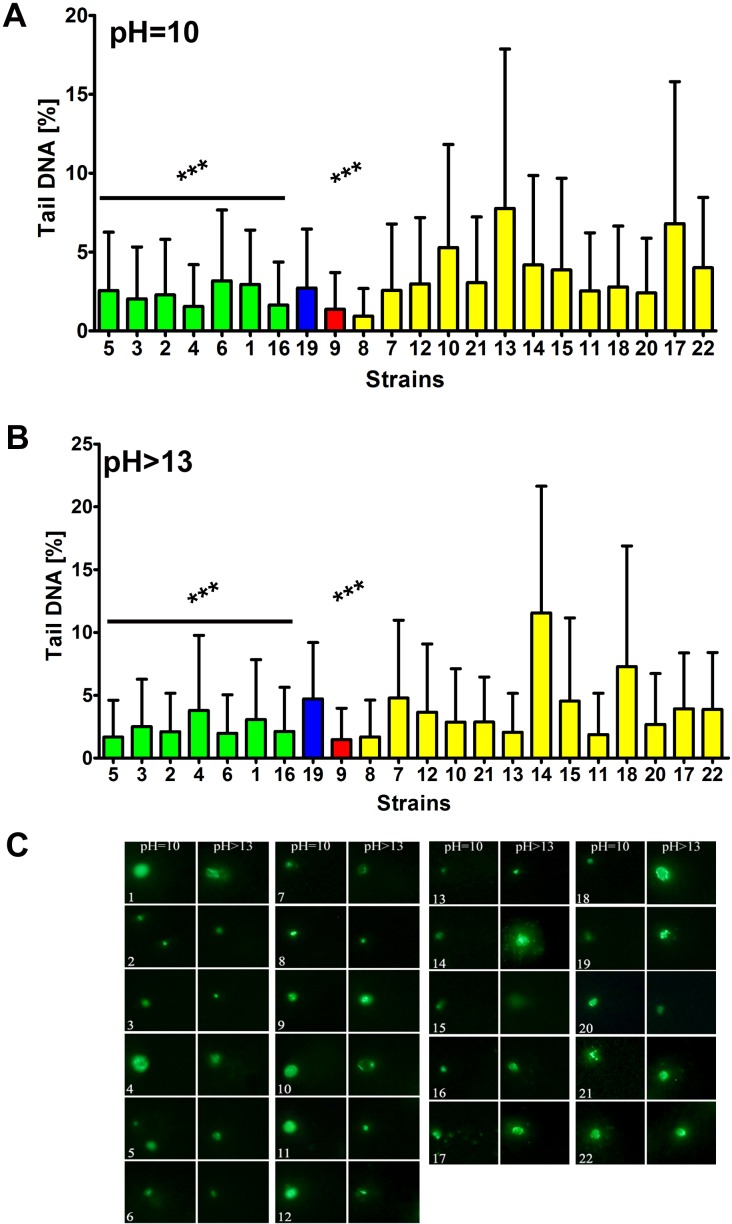
The susceptibility to DNA double strand breaks (DSBs) **A.** and DNA single strand breaks (SSBs) **B.** DSBs and SSBs were assessed using neutral and alkaline comet assay, respectively. The strains belonging to the same species were grouped together and the data were marked in different colors (*S. bayanus*: green, *S. kudriavzevii*: blue, *S. cerevisiae*: red and *S. paradoxus*: yellow). As a DNA damage marker, the % tail DNA was used. The bars indicate SD, *n* = 150, ^***^p < 0.001 compared to the *S. paradoxus* group (ANOVA and Tukey's a posteriori test). **C.** The typical micrographs are shown. DNA was visualized using YOYO-1 staining (green).

**Figure 8:**
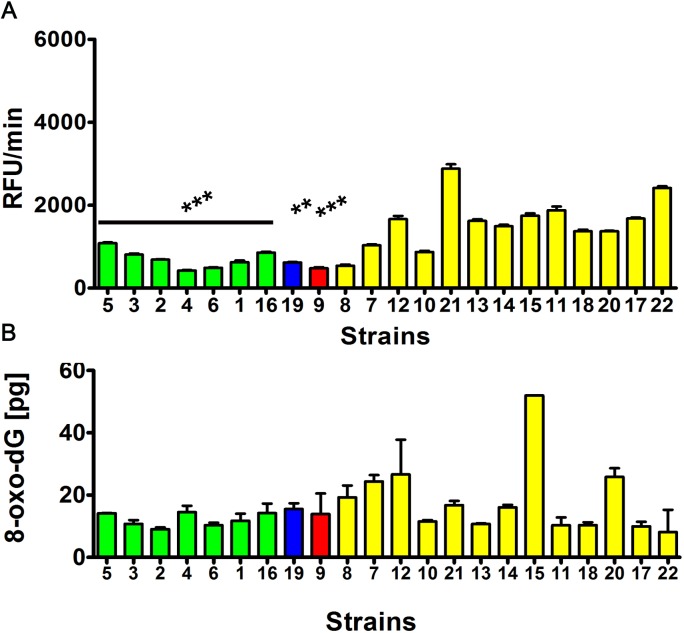
The intracellular reactive oxygen species (ROS) production **A.** and the level of oxidative DNA damage (8-hydroxy-2′-deoxyguanosine, 8-oxo-dG, level) **B.** ROS production was assessed using H_2_DCF-DA fluorogenic probe and the results are presented as relative fluorescence units per minute (RFU/min). The level of 8-oxo-dG was analyzed using ELISA-based assay. The strains belonging to the same species were grouped together and the data were marked in different colors (*S. bayanus*: green, *S. kudriavzevii*: blue, *S. cerevisiae*: red and *S. paradoxus*: yellow). The bars indicate SD, *n* = 5, ^***^p < 0.001, **p < 0.01 compared to the *S. paradoxus* group (ANOVA and Tukey's a posteriori test).

We hypothesized that altered Y' telomeric sequence-dependent helicase activity may modulate genetic stability in distillery strains. Thus, we also evaluated the strain-dependent susceptibility to DNA damage (Figures 7 and 8).

Indeed, the *S. bayanus* strain group (*p* < 0.001) and strains 9 (*S. cerevisiae*) (*p* < 0.001) and 19 (*S. kudriavzevii*) with the abundance of Y' telomeric sequences and higher number of *YRF1* gene copies were less affected by DNA double strand breaks (DSBs) and DNA single strand breaks (SSBs) than the *S. paradoxus* strain group (Figure 7). Moreover, the level of oxidative DNA damage (8-hydroxy-2′-deoxyguanosine, 8-oxo-dG, content) was increased in the *S. paradoxus* group compared to the *S. bayanus* group (Figure 8). However, the effect was statistically insignificant. The intracellular production of reactive oxygen species (ROS) was also elevated in the *S. paradoxus* group (*p* < 0.001) but no clear-cut relationship between ROS production and the 8-oxo-dG level was observed in this group, e.g., strains 21 and 22 with the most imbalanced redox equilibrium were characterized by relatively low level of 8-oxo-dG (Figure 8). Thus, it might not be concluded that the elevation in 8-oxo-dG level was a result of increased ROS production in the *S. paradoxus* group.

## DISCUSSION

This is the first report on detailed evaluation of genomic features of twenty two distillery yeast strains used in food industry to produce distilled spirits such as vodka and whisky. To date, one paper has been published on molecular genetic characteristics of thirty six distillery yeast strains belonging to the *S. cerevisiae* species [15]. The authors performed PCR-RFLP analysis of rDNA 5.8S-ITS fragment, molecular karyotyping (PFGE separation), and Southern blot-based detection of *MAL*, *SUC* and *MEL* genes [15]. Analyzed strains were aneuploid and rich in polymeric genes *SUC* and *MAL* important for sucrose and maltose fermentation, respectively [15]. As we have purchased the strains from multiple suppliers, we are aware that our analyzed “distillery group” may be more heterogeneous. Indeed, the strains examined in the present study were more diverse and belonged to four species of the *Saccharomyces sensu stricto* complex, namely *S. bayanus* (*n* = 7), *S. paradoxus* (*n* = 13), *S. cerevisiae* (*n* = 1) and *S. kudriavzevii* (*n* = 1) according to electrophoretic karyotyping. The obtained species-specific chromosome patterns were in agreement with previously reported data on karyotypic characteristics of reference yeast strains [9, 17–19]. Similar chromosome profiles were observed within the *S. bayanus* group (strains 1 to 6 and strain 16). However, one should remember that some karyotypic variants may also occur within the yeast species. This is particularly true for the *S. bayanus* group [6, 19]. *S. bayanus* var. *uvarum* isolates are typically characterized by only two small chromosomal bands in the range of 245-370 kb (between chromosomes I and III) instead of three or more in *S. bayanus* var. *bayanus* [6, 19]. The strains assigned to the *S. bayanus* group (this study) exhibited karyotypic features of the *S. bayanus* var. *bayanus.* In general, the analyzed strains were diploid but aneuploid events (the presence of some additional chromosome bands) were also observed. It is widely accepted the industrially relevant yeast strains, e.g., brewer's and wine yeasts, are aneuploid with disomies, trisomies and tetrasomies [20, 21]. Alloploidy is also a common phenomenon [1]. The best and most well-known example of industrial hybrid is the lager yeast *S. pastorianus* (syn. *S. carlsbergensis*), which is the cold-adapted *S. cerevisiae* x *S. eubayanus* allotetraploid [22]. Under certain conditions, e.g., during fermentation-associated biotic and abiotic stresses, aneuploidy events and changes in the ploidy may be adaptive and advantageous by increasing the number of copies of beneficial genes or by protecting the yeasts against recessive lethal or deleterious mutations that may confer resistance to low temperature or high ethanol levels [20, 23].

Genome-wide array-CGH analysis reveals variations in the gene copy number almost exclusively in the subtelomeric regions of the genome of distillery yeasts, and the most affected chromosomes were the chromosome I, III, VI and IX. It is worthwhile to note that the strain relatedness based on array-CGH data was comparable with electrophoretic karyotyping-based similarities among strains. Statistically significant differences in the gene dosage were observed in six functional gene categories, namely 1) telomere maintenance *via* recombination, DNA helicase activity or DNA binding, 2) maltose metabolism process, glucose transmembrane transporter activity; 3) asparagine catabolism, cellular response to nitrogen starvation, localized in cell wall-bounded periplasmic space, 4) siderophore transport, 5) response to copper ion, cadmium ion binding and 6) L-iditol 2- dehydrogenase activity. The effects were species-dependent that may suggest that strains within distillery group analyzed may differently respond to changing environments and may have diverse adaptation strategies. Surprisingly, in almost all gene categories, the effects observed in the *S. bayanus* and *S. paradoxus* groups were opposite, e.g., increased and decreased copy number of *YRF1* genes (*YRF1-1* to *YRF1-7*) in the *S. bayanus* and *S. paradoxus* group was shown, respectively. The *YRF1* genes (*YRF1-1* to *YRF1-7*) are localized on different yeast chromosomes within the Y' element of subtelomeric regions and encoded Y' element ATP-dependent helicase (Y'-Help1, Y'-HELicase Protein 1) implicated in telomerase-independent telomere maintenance [24]. In laboratory yeasts, Y'-Help1 is highly induced in the survivors of telomerase deficient cells [24]. It has been speculated that Y'-Help1 may enhance homologous DNA recombination among Y' elements and, as a consequence, may induce Y' amplification to prevent chromosomal loss and cell death [24]. We hypothesized that altered *YRF1* gene copy number and the presence of Y' elements may affect genetic stability in distillery strains. Indeed, the strains from the *S. paradoxus* group with decreased *YRF1* gene dosage and the lack of Y' sequences were more prone to DNA double and single strand breaks and oxidative DNA damage than the *S. bayanus* group that may influence the biotechnological processes using distillery strains. The opposite effect, namely increased copy number of *MEC3* gene encoded a DNA damage and meiotic pachytene checkpoint protein [25, 26] was observed in the *S. paradoxus* group that may have implications for DNA damage response and adaptations to DNA-damaging conditions.

The other genes with affected copy number were mainly involved in carbohydrate and amino acid metabolism, and ion transport that may also modulate a biotechnological process. The dosage of numerous genes implicated in maltose metabolism was affected (e.g., *MAL11*, *MAL13*, *MAL31*, *MAL33*, *MPH2* and *MPH3*). The *MAL* gene family of *Saccharomyces* is comprised of five multigene complexes, *MAL1*, *MAL2*, *MAL3*, *MAL4* and *MAL6*, located at or near the telomere of a different chromosome, any one of which is sufficient for yeast to metabolize the disaccharide maltose and encodes maltose permease (GENE l), maltase (GENE 2) and the *trans*-acting MAL-activator (GENE 3) [27]. *MAL11* and *MAL13* are part of the *MAL1* complex locus located on chromosome VII and encode high-affinity maltose transporter (α-glucoside transporter) and MAL-activator protein, respectively, whereas *MAL31* and *MAL33* are part of the *MAL3* complex located on chromosome II and encode maltose permease and MAL-activator protein, respectively [28, 29]. It has been suggested that the *MAL* loci have been translocated to different chromosomes *via* a mechanism that involved the rearrangement(s) of chromosome termini [30]. *MPH2* and *MPH3* genes (maltose permease homologs) encode α-glucoside permeases that transport maltose, maltotriose, α-methylglucoside, and turanose [31].

The distillery strains also differed in the copy number of *ASP3* genes, especially highly elevated *ASP3* gene copy number was revealed in strain 19 (*S. kudriavzevii*). *ASP3* contains a gene cluster located on chromosome XII comprised of four identical genes, *ASP3-1*, *ASP3-2*, *ASP3-3*, and *ASP3-4*, which encode for cell wall-associated L-asparaginase II that catalyzes the conversion of L-asparagine to aspartate and ammonia [32]. Asp3p is induced in response to nitrogen starvation and regulated by Gln3p/Ure2p [33]. More recently, the *ASP3* locus has been shown to be originated by horizontal gene transfer from *Wickerhamomyces* [34]. It has been speculated that *ASP3* acquisition may have aided yeast adaptation to artificial environments and may further highlight the importance of gene sharing between yeasts in the evolution of their remarkable metabolic diversity [34].

The most accented differences were observed in the copy number of *SOR1* and *SOR2* genes. The *SOR1* gene encode a NAD-dependent sorbitol dehydrogenase that is a member of the polyol dehydrogenase branch of the medium-chain dehydrogenase/reductase (MDR) superfamily of enzymes [35]. It has been reported that the expression of *SOR1* gene is elevated in the presence of sorbitol or xylose, though *S. cerevisiae* is a non-xylose-utilizing microorganism [35, 36]. Similarly, high variability in the gene copy number of genes involved in the siderophore transport, namely *ENB1*, *FRE3*, *FRE5*, *FIT2* and *FIT3*, was observed. They represent two genetically separable systems for the uptake of siderophore-bound iron in *S. cerevisiae*. One system is based on family of transporters that is expressed as part of the *AFT1* regulon and are termed *ARN1*, *ARN2* (*TAF1*), *ARN3* (*SIT1*) and *ARN4* (*ENB1*) [37, 38]. These transporters are expressed in intracellular vesicles [39]. The second system relies on the high affinity ferrous iron transport complex, which is encoded by *FET3* and *FTR1* and is located on the plasma membrane [40, 41]. Ferric reductases encoded by *FRE* genes take part in iron uptake by the reduction of siderophore-bound iron prior to uptake by transporters [42, 43]. There are also three cell wall mannoproteins (Fit1, Fit2, Fit3) that facilitate the uptake of iron [44]. Low iron levels stimulate the expression of components of both systems [45]. Perhaps, increased copy number of genes involved in the transport of siderophore-bound iron in the *S. paradoxus* group may be advantageous in the certain growth conditions, e.g., during iron deprivation. Additionally, in all groups analyzed, the metallothionein gene dosage *CUP1-1* and *CUP1-2* was increased that was the most accented in strain 9 (*S. cerevisiae*). This may be also beneficial as may confer resistance to copper and cadmium [46].

In conclusion, we have provided for the first time array-CGH-based comprehensive genomic characterization of commercially available twenty two distillery yeast strains. We have documented the naturally occurring diversity in the gene copy number within six functional gene categories and revealed that the variations in the *YRF1* gene copies may be accompanied by altered genetic stability in the analyzed yeast groups. Our genomic data may be helpful for better understanding of the fermentative environment-mediated changes in the yeast genome and accompanying phenotypic features. Thus, the knowledge on genetic diversity of distillery strains may be further exploited in economically important biotechnological processes.

## MATERIALS AND METHODS

### Reagents

All reagents, if not otherwise mentioned, were purchased from Sigma (Poland) and were of analytical grade.

### Yeast strains and growth conditions

All distillery yeast strains used in this study are listed in Table 1. Yeast from one single colony was grown either on liquid YPD medium (1% w/v Difco Yeast Extract, 2% w/v Difco Yeast Bacto-Peptone, 2% w/v dextrose) or on solid YPD medium containing 2% w/v Difco Bacto-agar, at 28°C.

### Pulsed-field gel electrophoresis (PFGE)

Preparation of agarose-embedded yeast DNA and PFGE separation of yeast DNA were conducted as described elsewhere [47]. The dendrogram of chromosomal DNA-based similarity was created using Free-Tree software [48] using UPGMA (Unweighted Pair Group Method with Arithmetic Mean) algorithm, Jaccard similarity coefficient and Java TreeView 1.1.6.r2 (http://jtreeview.sourceforge.net/).

### FACS-based ploidy analysis

The DNA content was measured *via* flow cytometry as previously described [49] except that a total of 3×10^4^ cells were counted in a single assay.

### Array-based comparative genomic hybridization (array-CGH)

Genomic DNA (0.5 μg) was labeled with SureTag DNA Labeling Kit and either Cy3- or Cy5-dUTP. Equal amounts of labeled DNA of tested and of the reference strain (BY4741) were combined and hybridized to Yeast (V2) Gene Expression Microarray, 8x15K using Oligo aCGH Hybridization Kit. All components were supplied by Agilent Technologies Inc. (Santa Clara, CA, USA) and all steps of the experiment were performed according to manufacturer's protocols. Following hybridization and washing, the slides were scanned using Axon GenePix 4000B. Feature extraction was conducted using GenePix Pro 6.1 and normalization using Acuity 4.0 (Molecular Devices, Sunnyvale, CA, USA). CGH profiles with superimposed piecewise regression plots to highlight aneuploidies, were generated using CGH-Explorer v3.2 [50]. The original CGH profiles obtained after the comparison of analyzed strains to BY4741 gave consistently high noise due most probably to genomic DNA sequence differences between BY4741 and the industrial strains, which influenced the hybridization strength of individual probes. Therefore to obtain final CGH profiles, the data for each strain were compared to the average of all industrial strains used in the experiment.

### Gene analysis after array-CGH

The analysis of over-representation of functional categories was performed using Cytoscape v. 2.8.2 with BiNGO v. 2.44 plug-in and hypergeometric test using Benjamini and Hochberg False Discovery Rate (FDR) correction and significance level of 0.05.

### Cluster analysis

The array-CGH data for all strains were subjected to complete linkage clustering with Cluster 3.0 software using Euclidean distance similarity metrics [51]. To obtain the tree graph of similarity, the clustering output was visualized using Java TreeView 1.1.6.r2 (http://jtreeview.sourceforge.net/).

### Detection of telomeric Y' sequences

Y' element telomeric probe was obtained according to [52] with minor modifications. After standard PFGE separation, Y' sequences within particular yeast chromosomes were detected using digoxigenin labeling, anti-digoxigenin antibody and phosphate alkaline-based chemiluminescence [53].

### Comet assay

Yeast spheroplasts were obtained [47] and DNA double-strand breaks (DSBs) and DNA single-strand breaks (SSBs) were assessed by neutral and alkaline single-cell microgel electrophoresis (comet assay), respectively, as described elsewhere [54]. The percentage of tail DNA was used as a parameter of DNA damage.

### Oxidative stress parameters

Intracellular reactive oxygen species (ROS) production was measured using 2′,7′-dichlorodihydrofluorescein diacetate (H_2_DCF-DA) as described elsewhere [53]. Oxidative DNA damage as a level of 8-hydroxy-2′-deoxyguanosine (8-OHdG, 8-oxo-dG) was measured using Epigentek EpiQuik 8-OHdG DNA Damage Quantification Direct Kit (Gentaur, Poland) using the standard protocol according to the manufacturer's instructions.

### Statistical analysis

The results represent the mean ± SD from at least three independent experiments. Statistical significance was assessed by 1-way ANOVA using GraphPad Prism 5, and with the Tukey's multiple comparison test.

## SUPPLEMENTARY FILES


